# Purification of Capsular Polysaccharides of *Streptococcus pneumoniae*: Traditional and New Methods

**DOI:** 10.3389/fbioe.2018.00145

**Published:** 2018-10-12

**Authors:** Victor Morais, Valerie Dee, Norma Suárez

**Affiliations:** Department of Biotechnology, Institute of Hygiene, Faculty of Medicine, University of the Republic, Montevideo, Uruguay

**Keywords:** capsular polysaccharides, *Streptococcus pneumoniae*, purification, ethanol precipitation, vaccine

## Abstract

Pneumonia caused by *Streptococcus pneumoniae* is a major bacterial disease responsible for many deaths worldwide each year and is particularly dangerous in children under 5 years old and adults over 50. The capsular polysaccharide (CPS) constitutes the outermost layer of the bacterial cell and is the main virulence factor. Regardless of whether pharmaceutical agents are composed of CPS alone or protein-conjugated CPS, CPS purification is essential for the development of vaccines against *S. pneumoniae*. These vaccines are effective and safe but remain quite expensive. This review describes the methods currently available for CPS purification. Advances in CPS purification methods are aimed at improvements in quality and yield and, above all, process simplification.

## Introduction

*Streptococcus pneumoniae* is a pathogen that was first isolated by Louis Pasteur and George Sternbern, independently, in 1880 (Grabenstein and Klugman, 2012). Years later, this bacteria was recognized as the main agent causing pneumonia and as being capable of producing meningitis, otitis media, and other infectious diseases. In many underdeveloped countries, pneumonia caused by *S. pneumoniae* is the bacterial disease responsible for the major proportion of deaths in children under 5 years of age and adults over 50 (Blasi et al., 2012).

*S. pneumoniae* exclusively infects humans, with its ecological niche being the nasopharynx since there is no other reservoir in nature. The route of transmission of pneumococcus is through saliva droplets from carriers or patients. Pneumococcus is an organism sensitive to heat, cold, and desiccation; therefore, transmission requires close contact from person to person. It is characterized by the frequency with which it colonizes and by the time it can remain in the nasopharynx without causing disease (Marchisio et al., 2002; Wattal et al., 2007). Carriers may harbor different serotypes simultaneously or at different times, either continuously or intermittently (Lipsitch et al., 2005).

*S. pneumoniae* are encapsulated, aerotolerant anaerobic, gram-positive bacteria (Auzat et al., 1999). They are immobile, non-sporulating and capable of employing a wide variety of carbohydrates as carbon sources (Bidossi et al., 2012). Microscopically, *S. pneumoniae* appear as lanceolate diplococci, frequently grouped into short chains, while macroscopically, they present as bright, α-hemolytic, circular colonies. The capsular polysaccharide (CPS) constitutes the outermost layer of the bacterial cell and is the main virulence factor (Martens et al., 2004).

This pathogen has a large number of different immunological types, differentiated by their CPS. Approximately 97 different specific types have been identified (Geno et al., 2015). There are two types of classification systems based on serological relationships: the American system (Henrichsen, 1995) and the Danish system, (Lund, 1963).

In addition to the CPS, *S. pneumoniae* have other virulence factors that act in the disintegration and lysis of the pneumococcal bacteria (autolysin), activating complements and enhancing inflammation (peptidoglycan, cell-wall polysaccharides, and pneumolysin), inhibiting complement activation (PspA and PspC), etc. (Mitchell et al., 1991; Neeleman et al., 1999). Recent sera screening of a collection of pneumonia patients permitted the identification of two new pneumococcus surface proteins as potential antigens, namely, PcsB, a protein required for cell wall separation, and StkP, a serine-threonine protein kinase. Both are highly conserved proteins (Daniels et al., 2016).

Although *S*. *pneumoniae* is considered to be mainly susceptible to penicillin and other antimicrobial agents, the emergence of resistant strains worldwide has made it difficult to treat diseases caused by this aetiological agent (Kim et al., 2016). Adults and children over 5 years of age are able to develop immunity against *S*. *pneumonia*, either via a previous infection or vaccination. Immunity developed in this way confers protection against subsequent infections (Koskela, 1987). The diversity of serotypes and serotype replacement constitute the main challenges to developing more effective vaccines (Daniels et al., 2016; Balsells et al., 2017). Even if a vaccine is successfully developed for a specific region, subsequent replacement of vaccine-targeted serotypes with non-vaccine serotypes may occur, minimizing the coverage and effectiveness of the vaccine with time (Geno et al., 2015).

## CPS composition

The capsular polysaccharides of *S. pneumoniae* were isolated in 1916 by Dochez and Avery. Some years later, capsular antigens were established as the basis of the serotypes of pneumococcal bacteria (Grabenstein and Klugman, 2012). In the 1930s, the critical immunogenic role of pneumococcal CPS as a virulence factor was established (Grabenstein and Klugman, 2012).

The pneumococcal CPS capsule is a surface structure consisting of mucous or viscous material, located external to the cell wall. It protects the pathogen from host defense mechanisms (Alonso et al., 1995). The capsular material is composed of a high-molecular-weight polymer made up of repeating units of oligosaccharides bound by covalent bonds to the cell wall (Yother, 2011). The virulence and invasiveness of pneumococcal strains vary according to the serotypes and depend on the chemical composition and amount of CPS produced (Zafar et al., 2017). These differences determine the survival of the bacteria once in circulation and the possibilities for causing invasive disease (Watson and Musher, 1990). Several capsules are highly polar and hydrophilic, thus interfering with the interactions between the bacteria and phagocytes. Chemical structure studies of this antigen reveal that most types contain a negatively charged capsule (except for serotypes 7 and 14, which are neutral) and acid components, such as glucuronic acid (in types 1, 2, 3, 5, 8, 9A, and 9V), or phosphate in phosphodiester bonds (in types 6A, 6B, 11A, 15F, 19F, 19A, and 23F) (Jansson et al., 1985).

The capsule is synthesized rapidly and extensively during the logarithmic phase of bacterial growth by similar mechanisms in most *Streptococcus* species. In *S. pneumoniae*, biosynthesis is believed to occur through the formation of repetitive units attached to a carrier lipid, which is synthesized on the intracellular side of the membrane. The altered carrier lipid is then exported to the surface, where the repeating units of oligosaccharides are polymerised (Kolkman et al., 1997; Yother, 2011). At some point during polymerization, the capsule is covalently attached to the cell wall, presumably by linkage via the reducing-end glucose of the CPS and the β-D-N-acetylglucosamine residues of peptidoglycan (Larson and Yother, 2017). This general organization has been identified in the capsule loci of all known serotypes except for serotypes 3 and 37, which are synthesized by a different mechanism, which is independent of the lipid carrier (Cartee et al., 2000; Geno et al., 2015). Capsule genes are organized as cassettes containing regions of genes that encode the functions required for production of specific capsular structures and are bound by other gene regions common to capsules of all serotypes (Bentley et al., 2006; Wu et al., 2016). These common regions found in almost all capsule types comprise 4 genes, cpsA, cpsB, cpsC, and cpsD (Guidolin et al., 1994; Morona et al., 1997), which encode the proteins necessary for production or regulation of the capsule. The structural properties of each CPS (such as the functional groups on a hydrocarbon backbone) or the number of polymer chains are key factors determining biological function and purification strategy (Cartee et al., 2000; Gonçalves et al., 2007).

The importance of CPS purification is related to the development of a new generation of *S. pneumoniae* vaccines composed of CPS alone or conjugated to proteins. Schiemann and Casper discovered the immunogenicity of CPS (Grabenstein and Klugman, 2012) in 1927. Several years later, Avery improved immunogenicity by covalently coupling CPS to proteins, and Felton showed that pure pneumococcal polysaccharides induced the production of antibodies in humans (Grabenstein and Klugman, 2012). It would take many decades for this development to attain practical clinical value.

## Methods of purification

Separation and purification procedures for polymers have been developed and applied to many important fields, such as manufacturing, pharmaceuticals and medicine, due to their interesting biological activities. The high purities required for polysaccharides specific to medical and drug manufacturing have led to the development of new purification methods based on fractional precipitation, ion exchange chromatography, gel filtration, and affinity chromatography (Khodakarami and Alagha, 2017; Li et al., 2017).

Several articles or patents pertaining to *S. pneumoniae* CPS purification have been published. These generally describe modifications to upstream procedures that facilitate subsequent purification. After fermentation of the desired serotype, most authors collect cell cultures by centrifugation and lyse the cells using sodium deoxycholate (Jung et al., 2011). Other authors have added sodium deoxycholate after fermentation but before collecting cells and sourced CPS from the supernatant or filtrate (Macha et al., 2014). Zanardo and coworkers prefer to inactivate the bacteria with thimerosal and remove cells by filtration because lysing with detergent breaks down cell membranes, releasing large amounts of intracellular contaminants that make the purification process difficult (Zanardo et al., 2016). The main contaminants requiring separation include proteins, nucleic acids and cell-wall carbohydrates (C-Carbohydrates). According to the World Health Organization, the desirable amounts of protein and nucleic acid contaminants are < 3 and 2%, respectively (WHO, 2009). The first patents related to CPS purification coincided with the development and production of the first vaccines against carbohydrate antigens of the capsule. Most CPS purification processes include one or more ethanol precipitations. The CPS types are named according to the Danish system (Lund, 1963).

**Figure 1:**
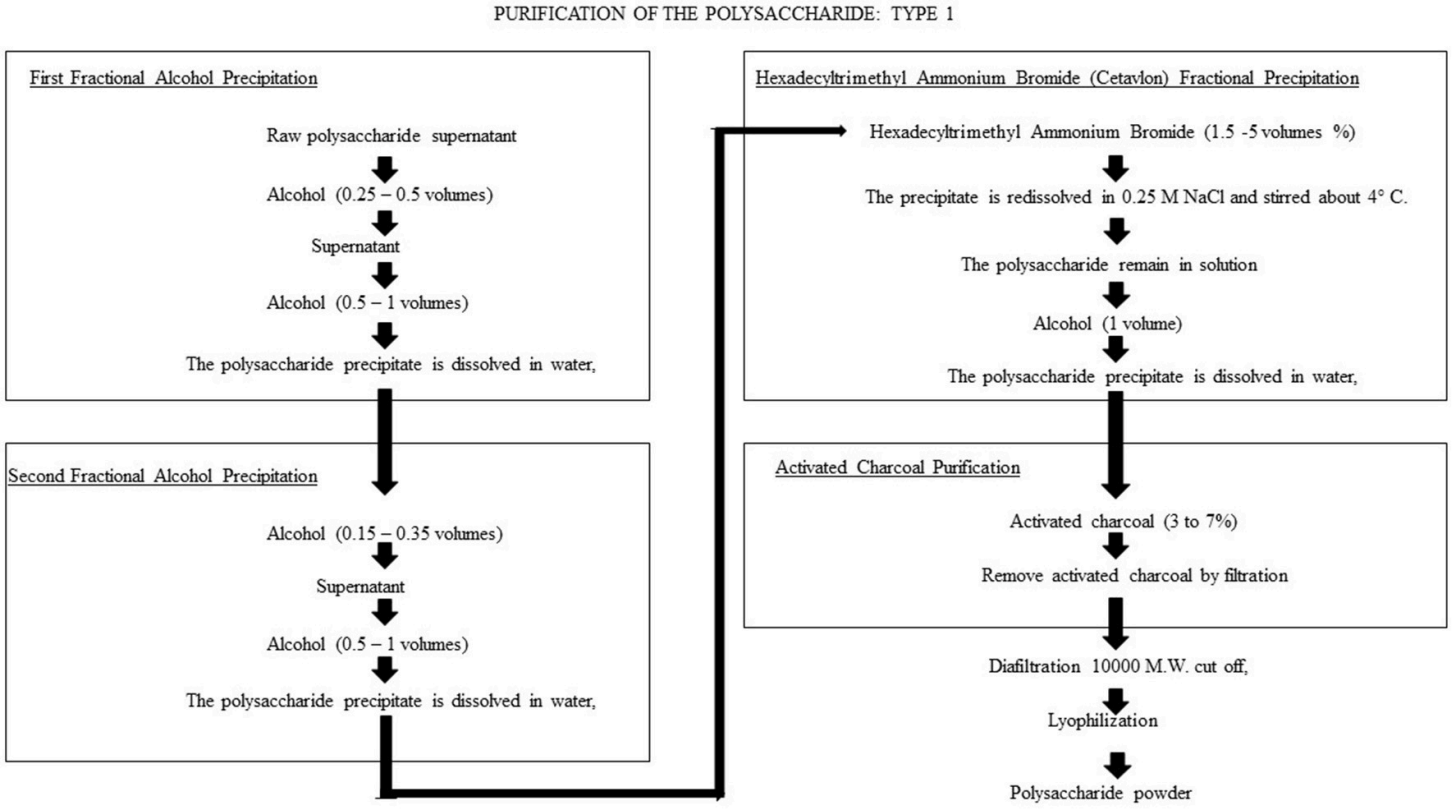
Flow diagram for the CPS type-1 purification process proposed by Cano et al. (1979). The purification method includes ethanol precipitation, hexadecyltrimethylammonium bromide (Cetavlon) precipitation and purification by activated charcoal. The process removed more than 99% of contaminant protein, nucleic acid and C-carbohydrates while retaining the immunogenicity of the product.

Figure 1 shows the flow diagram for purification of CPS type 1, as proposed by Cano et al. (1979). The general process is suitable for 16 pneumococcal capsular polysaccharide types (1, 2, 3, 4, 6A, 6B, 7F, 8, 9N, 12F, 14, 18C, 19F, 20, 23F, and 25), with some modifications between types. In general, all purification methods include ethanol precipitation, hexadecyltrimethylammonium bromide (Cetavlon) precipitation and purification using activated charcoal. For some CPS types, precipitation with ammonium sulfate is also included. The authors reported that the processes removed more than 99% of contaminant proteins, nucleic acids and C-carbohydrates while retaining the immunogenicity of the product. Many of the procedural steps are complex and time consuming; for example, the Cetavlon elimination requires extensive washing to remove all the residues. Unfortunately, despite the number of recorded patents, no results for yields or final purities were reported.

In the same year, a patent presented by Merck Company (Carlo et al., 1979) described a polysaccharide purification process that includes ethanol, isopropanol, and cetrimonium bromide precipitations, protease and nuclease treatments and, if necessary, dialysis to remove any remaining contaminants. From 14 l of culture, these authors could recover from 0.35 g (type 1) to 4.6 g (type 2) of purified CPS.

In the 1980s, the Merieux Institute patented another classical polysaccharide purification process for *S. pneumoniae* (Merieux et al., 1981). The patent describes the purification of 17 CPS of *S. pneumoniae*. Applying a semi-synthetic medium to an industrial culture of *S. pneumonia*, the purification process includes ethanol precipitation, phenol extraction, and purification using activated charcoal, with some variation depending on CPS type. The purity and yield vary depending on the CPS type and initial culture volume but are similar to those reported in patents recorded previously. For example, recoveries of 0.4, 0.3, and 0.1 g/l were obtained for CPS types 1, 2, and 4, respectively. It is worth noting that this patent utilizes a semi-synthetic medium, underscoring the importance of the culture media in determining the polysaccharide purity at the end of the process.

In 1998, Arnold et al. patented a purification method without ethanol precipitation (Arnold and Soika, 1998) for 23 CPS types (1, 2, 3, 4, 5, 6B, 7F, 8, 9N, 9V, 10A, 11A, 12F, 14, 15B, 17F, 18C, 19A, 19F, 20, 22F, 23F, and 33F). The process includes hexadecyltrimethylammonium bromide precipitation and hydroxyapatite (HA) chromatography. The authors found that all pneumococcal polysaccharide types precipitated with 1–4%, with the exception of pneumococcal CPS types 7F, 14, and 33F. Because precipitation with hexadecyl trimethyl ammonium is based on interactions between positive charges on the detergent and negative charges on the CPS, CPS types 7F, 14, and 33F do not precipitate due to their neutral features; therefore, an additional Q-Anion Exchange column chromatography step was added. The authors reported removal of 95% of both protein and nucleic-acid contaminants, compared to crude lysate values, but did not report final yields or purities.

In general, these classical processes highlight the importance of eliminating contaminants and the disadvantages of using certain toxic reagents, such as phenol (during the process) or DOC (for cell lysis), which release abundant intracellular contaminants into the extracellular media. As a consequence, the addition of new purification steps to eliminate these contaminants increases the complexity of the process, decreasing the final yields and increasing the economic costs.

New methods for capsule purification have been tested to attain better yields and purity. Although some tedious steps have been eliminated or replaced, a multistep procedure is still in use. Recently, new purification methods have been developed, aiming to attain higher CPS purity and yield with a simplified workflow (Table 1). Most of these are specific to only one CPS type, but they could be adapted for other types with similar physicochemical properties.

**Table 1:** CPS purification processes.

**Process**	**Number of steps**	**Main separation process**	**Polishing process**	**Main advantages**	**Main disadvantages**
Traditional methods	>10	Ethanol precipitation Cetavlon precipitation Phenol extraction	Purification by activated charcoal Ammonium sulfate precipitation	Optimized for many serotypes	Many steps Low yields
Suárez et al., 2001	2	Affinity chromatography		High purity Few steps	Difficult scale up Optimized for only 1 serotype (14)
Gonçalves et al., 2007	7	Ultrafiltration Ethanol precipitation	Protease and nuclease treatment	Good recovery	Enzyme step addition Optimized for only 2 serotypes (6B, 23F)
Jung et al., 2011	4	pH precipitation Ethanol precipitation		Good recovery Few steps	Optimized for only 1 serotype (19A)
Macha et al., 2014	7	Ultrafiltration Ethanol precipitation	Aluminum phosphate co-precipitation	Good recovery Endotoxin elimination	Optimized for only 5 serotypes
Zanardo et al., 2016	6–7	Ultrafiltration TCA precipitation Ethanol precipitation	Anion exchange chromatography (if necessary)	Good yield Simple method Easily scale-up	Optimized for only 1 serotype (14)

*Advantages and disadvantages of new and traditional methods*.

Recently, Gonçalves et al. (2003, 2007) proposed a process for purifying the 23F serotype that consists of concentration by tangential ultrafiltration (30 kDa), fractional ethanol precipitation (28–60%), nuclease and proteinase treatment, and concentration/diafiltration via a 30-kDa cassette. The authors achieved final polysaccharide recoveries of 89%, with protein and nucleotide contamination of < 2%. Interestingly, these authors also developed a new batch cultivation process for high polysaccharide production of this serotype using the supernatant fraction, thereby avoiding the use of the whole culture (Gonçalves et al., 2002). The process allows large-scale CPS production and simplification of the downstream process.

Some years later, Jung et al. (2011) proposed a simplified purification procedure for serotype 19A, which included adjusting the cell lysate pH to 4.5, fractionation with 50–80% ethanol, and finally dialysis. The CPS process was completed in < 48 h, a significantly shorter time period compared with other published procedures. The authors attained a yield of 75% and a purity of 97% at the end of the process. The use of an acidic pH to precipitate soluble proteins or other soluble components at the beginning of the purification process significantly improved the purity of the final product; however, this procedure is only applicable to acid-resistant polysaccharides.

Macha and colleagues proposed a simplified purification method for CPS types 3, 6B, 14, 19F, and 23F that utilizes lysis by detergent, followed by concentration, ethanol precipitation, aluminum phosphate adsorption and diafiltration with a 300-kDa tangential flow filtration system. The resulting percentage of process impurities was < 1.5%, and the recovery was 65–80% (Macha et al., 2014). The advantage of this method is that it replaces phenol precipitation and the use of nucleases and proteases with AlPO_4_ adsorption and ultrafiltration. The authors reported that AlPO_4_ is capable of separating two negatively charged compounds (for example, CPS and endotoxin) by selectively binding to endotoxins and separating them from the CPS.

Furthermore, Zanardo and coworkers evaluated a new purification process for serotype 14 (Zanardo et al., 2016), assaying three purification strategies consisting of the following steps: cell separation by tangential microfiltration, concentration by tangential ultrafiltration (50 kDa), diafiltration in the presence of sodium dodecyl sulfate using a 30-kDa ultrafiltration membrane, precipitation with 5% trichloroacetic acid, precipitation with 20 and 60% ethanol, and anion exchange chromatography. All processes achieved the required purity regarding nucleic acids (≤ 2%) and proteins (≤ 3%). Final CPS recoveries were between 35 and 60%.

A single step method by affinity chromatography (lectins) was established by Suárez et al. (2001) at the lab scale for serotype 14. The technique has advantages over others of an easy purification procedure and of being faster than traditional multistep methods. These authors achieved purity values above 99%, which are considerably high given that deoxycholate was used to lysate the cells. Nevertheless, the capacity of the method was limited to several milligrams of CPS, and scaling up the procedure could be quite expensive.

## Conclusion

Purification of CPS is essential for preparing vaccines against *S. pneumoniae*, regardless of whether these vaccines are composed of CPS alone or CPS conjugated to proteins. Such vaccines are effective and safe but remain quite expensive. Advances in CPS purification methods are geared toward improving quality and yield and simplifying the process.

While some changes in the upstream process have resulted in simplification of the purification process, the cellular lysis step breaks down cell membranes and releases intracellular contaminants. Thus, Zanardo and coworkers preferred to inactivate bacteria using thimerosal and remove cells by filtration (Zanardo et al., 2016). The use of continuous cultivations and chemically defined media also contributes to the simplification of the downstream process (Zanardo et al., 2016). Optimizing CPS production by adjusting the growth-media composition and culture conditions remains an important research area to improve the yields and purities of polysaccharide products (Zhang and Robinson, 2005; Marthos et al., 2015; Morais and Suárez, 2016).

It is desirable that the CPS for vaccine production be as homogeneous as possible in terms of chemical composition and molecular weight. The role of the culture medium in the synthesis of CPS remains an interesting direction for future studies.

In general, the CPS purification processes developed for *S. pneumoniae, Haemophilus influenza* type b, and *Neisseria meningitides* in recent decades have achieved increased efficiency by reducing the number of ethanol precipitations employed and eliminating the use of phenol (Suárez et al., 2001; Pato et al., 2006; Gonçalves et al., 2007; Albani et al., 2012, 2015; Sharma et al., 2015). Particularly for *Neisseria meningitidis*, Tanizaki et al. (1996) proposed a modification of the classical purification process for meningococcal group C polysaccharides by substituting the phenol extraction step by digestions with proteases and extensive diafiltration. Pato et al. (2006) reported a new approach including continuous centrifugation of the supernatant, tangential filtration, anion exchange chromatography, and size exclusion chromatography that improves the yield and quality of the polysaccharide.

Recently, Sharma and collaborators attempted to simplify the purification process of bacterial polysaccharides for serogroups A and C of *N. meningitidis* by utilizing O-acetylation and hydrophobic interaction chromatography (HIC), with good results (Sharma et al., 2015).

In the case of the *Haemophilus influenzae* type b (Hib) capsular polysaccharide, Albani et al. (2012) reported a purification procedure based on tangential ultrafiltration, ethanol precipitations, and enzymatic hydrolysis. This strategy eliminates phenol and reduces the number of ethanol precipitation steps. Later, the authors proposed a modification of the process by introducing tangential microfiltration to replace centrifugation, resulting in an improvement in the quality of the polysaccharide. In summary, all the process modifications proposed by these authors could be explored for pneumococcal CPS purification.

Particularly for *S. pneumoniae*, downstream purification procedures tend to use ethanol precipitation plus a polishing step (chromatography, ultrafiltration, TCA precipitation, and others). The use of different procedures depends on variabilities in serotypes as well as the respective CPS properties and the raw material utilized in the upstream process. While this review summarizes the most notable research in the development of capsular purification processes for *S. pneumoniae*, more studies are needed in the field of pneumococcal production for vaccine use.

## Author contributions

VM and NS participated in the conception, information search and assisted in drafting the manuscript. VD assisted in drafting and correction of the manuscript.

### Conflict of interest statement

The authors declare that the research was conducted in the absence of any commercial or financial relationships that could be construed as a potential conflict of interest.

## References

[B1] Albani S. M. F. Da Silva M. R. Fratelli F. Junior C. P. C. Iourtov D. Cintra F. D. O.. (2015). Polysaccharide purification from *Haemophilus influenzae* type b through tangential microfiltration. Carbohydr. Polym. 116, 67–73. 10.1016/j.carbpol.2014.03.046 25458274

[B2] Albani S. M. F. Da Silva M. R. Takagi M. Cabrera-Crespo J. (2012). Improvement in the purification process of the capsular polysaccharide from *Haemophilus influenzae* type b by using tangential ultrafiltration and diafiltration. Appl. Biochem. Biotechnol. 167, 2068–2075. 10.1007/s12010-012-9750-4 22665219

[B3] Alonso D. E. Verheul A. F. Verhoef J. Snippe H. (1995). *Streptococcus pneumoniae*: virulence factors, pathogenesis, and vaccines. Microbiol. Rev. 59, 591–603. 8531887 10.1128/mr.59.4.591-603.1995 PMC239389

[B4] Arnold F. Soika M. (1998). Alcohol-free pneumococcal polysaccharide purification process. Patent US 5,714,354. Available online at: https://patents.google.com/patent/US5714354A/en

[B5] Auzat I. Chapuy-Regaud S. Le Bras G. Dos Santos D. Ogunniyi A. D. Le Thomas I.. (1999). The NADH oxidase of *Streptococcus pneumoniae* : its involvement in competence and virulence. Mol. Microbiol. 34, 1018–1028. 10.1046/j.1365-2958.1999.01663.x 10594826

[B6] Balsells E. Guillot L. Nair H. Kyaw M. H. (2017). Serotype distribution of *Streptococcus pneumoniae* causing invasive disease in children in the post-PCV era: a systematic review and meta-analysis. PLoS ONE 12:e0177113. 10.1371/journal.pone.0177113 28486544 PMC5423631

[B7] Bentley S. D. Aanensen D. M. Mavroidi A. Saunders D. Rabbinowitsch E. Collins M.. (2006). Genetic analysis of the capsular biosynthetic locus from all 90 pneumococcal serotypes. PLoS Genet. 2:e31. 10.1371/journal.pgen.0020031 16532061 PMC1391919

[B8] Bidossi A. Mulas L. Decorosi F. Colomba L. Ricci S. Pozzi G.. (2012). A functional genomics approach to establish the complement of carbohydrate transporters in *Streptococcus pneumoniae*. PLoS ONE 7:e33320. 10.1371/journal.pone.0033320 22428019 PMC3302838

[B9] Blasi F. Mantero M. Santus P. Tarsia P. (2012). Understanding the burden of pneumococcal disease in adults. Clin. Microbiol. Infect. 18, 7–14. 10.1111/j.1469-0691.2012.03937.x 22882668

[B10] Cano F. Kuo J. Querry M. (1979). Purification of pneumococcal capsular polysaccharides. Patent US 4,242,501. Available online at: https://patents.google.com/patent/US4242501A/en

[B11] Carlo D. J. Nostadt K. H. Stoudt T. H. Walton R. B. Zeltner J. Y. (1979). Pneumococcal vaccine and a process for its preparation. Patent EP0002404A1. https://patents.google.com/patent/EP0002404A1/en

[B12] Cartee R. T. Forsee W. T. Schutzbach J. S. Yother J. (2000). Mechanism of type 3 capsular polysaccharide synthesis in *Streptococcus pneumoniae*. J. Biol. Chem. 275, 3907–3914. 10.1074/jbc.275.6.3907 10660543

[B13] Daniels C. C. Rogers P. D. Shelton C. M. (2016). A review of pneumococcal vaccines: current polysaccharide vaccine recommendations and future protein antigens. J. Pediatr. Pharmacol. Ther. 2721, 27–35. 10.5863/1551-6776-21.1.27 26997927 PMC4778694

[B14] Geno K. A. Gilbert G. L. Song J. Y. Skovsted I. C. Klugman K. P. Jones C.. (2015). Pneumococcal capsules and their types: past, present, and future. Clin. Microbiol. Rev. 28, 871–899. 10.1128/CMR.00024-15 26085553 PMC4475641

[B15] Gonçalves V. Takagi M. Carmo T. (2007). Simple and efficient method of bacterial polysaccharides purification for vaccines production using hydrolytic enzymes and tangential flow ultrafiltration, in Communicating Current Research and Educational Topics and Trends in Applied Microbiology, 450–457. Available online at: http://www.formatex.org/microbio/pdf/Pages450-457.pdf

[B16] Gonçalves V. M. Zangirolami T. C. Giordano R. L. C. Raw I. Tanizaki M. M. Giordano R. C. (2002). Optimization of medium and cultivation conditions for capsular polysaccharide production by *Streptococcus pneumoniae* serotype 23F. Appl. Microbiol. Biotechnol. 59, 713–717. 10.1007/s00253-002-1075-8 12226729

[B17] Gonçalves V. M. M. Takagi M. Lima R. B. Massaldi H. Giordano R. C. Tanizaki M. M. (2003). Purification of capsular polysaccharide from *Streptococcus pneumoniae* serotype 23F by a procedure suitable for scale-up. Biotechnol. Appl. Biochem. 37, 283–287. 10.1042/BA20020075 12515577

[B18] Grabenstein J. D. Klugman K. P. (2012). A century of pneumococcal vaccination research in humans. Clin. Microbiol. Infect. 18, 15–24. 10.1111/j.1469-0691.2012.03943.x 22882735

[B19] Guidolin A. Morona J. K. Morona R. Hansman D. Paton J. C. (1994). Nucleotide sequence analysis of genes essential for capsular polysaccharide biosynthesis in *Streptococcus pneumoniae* type 19F. Infect. Immun. 62, 5384–5396. 7960118 10.1128/iai.62.12.5384-5396.1994 PMC303279

[B20] Henrichsen J. (1995). Six newly recognized types of *Streptococcus pneumoniae*. J. Clin. Microbiol. 33, 2759–2762. 8567920 10.1128/jcm.33.10.2759-2762.1995 PMC228570

[B21] Jansson P.-E. Lindberg B. Lindquist U. (1985). Structural studies of the capsular polysaccharide from *Streptococcus pneumoniae* type 5. Carbohydr. Res. 140, 101–110. 10.1016/0008-6215(85)85053-9 4053092

[B22] Jung S. J. Seo E. S. Yun S.-Il. Minh B. N. Jin S.D Ryu H. J.. (2011). Purification of capsular polysaccharide produced by *Streptococcus pneumoniae* serotype 19A. J. Microbiol. Biotechnol. 21, 734–738. 10.4014/jmb.1010.10043 21791960

[B23] Khodakarami M. Alagha L. (2017). High-performance polymers for separation and purification processes: an overview. Polym. Plast. Technol. Eng. 56, 2019–2042. 10.1080/03602559.2017.1298800

[B24] Kim L. McGee L. Tomczyk S. Beall B. (2016). Biological and epidemiological features of antibiotic-resistant *Streptococcus pneumoniae* in pre- and post-conjugate vaccine eras: a United States perspective. Clin. Microbiol. Rev. 29, 525–552. 10.1128/CMR.00058-15 27076637 PMC4861989

[B25] Kolkman M. A. B. Van Der Zeijst B. A. M. Nuijten P. J. M. (1997). Functional analysis of glycosyltransferases encoded by the capsular polysaccharide biosynthesis locus of *Streptococcus pneumoniae* serotype 14. J. Biol. Chem. 272, 19502–19508. 10.1074/jbc.272.31.19502 9235953

[B26] Koskela M. (1987). Serum antibodies to pneumococcal C polysaccharide in children: response to acute pneumococcal otitis media or to vaccination. Pediatr. Infect. Dis. J. 6, 519–526. 3615065 10.1097/00006454-198706000-00006

[B27] Larson T. R. Yother J. (2017). *Streptococcus pneumoniae* capsular polysaccharide is linked to peptidoglycan via a direct glycosidic bond to β-D- *N*-acetylglucosamine. Proc. Natl. Acad. Sci. U.S.A. 114, 5695–5700. 10.1073/pnas.1620431114 28495967 PMC5465879

[B28] Li Z. Chen A. Li Z. Qu M. Chen H. Yang B. (2017). A novel and environmentally friendly bioprocess for separation and partial purification of polysaccharides from Cordyceps sinensis mycelia by an aqueous two-phase system. RSC Adv. 7, 37659–37665. 10.1039/C7RA05360F

[B29] Lipsitch M. Whitney C. G. Zell E. Kaijalainen T. Dagan R. Malley R. (2005). Are anticapsular antibodies the primary mechanism of protection against invasive pneumococcal disease? PLoS Med. 2:e15. 10.1371/journal.pmed.0020015 15696204 PMC545206

[B30] Lund E. (1963). Polyvalent, diagnostic pneumococcus sera. Acta Pathol. Microbiol. Scand. 59, 533–536. 10.1111/j.1699-0463.1963.tb01256.x 14080486

[B31] Macha C. Lavanya A. Nanna R. (2014). Purification of streptococcus pneumoniae capsular polysaccharides using aluminium phosphate and ethanol. Int. J. Pharm. Pharm. Sci. 6, 385–387.

[B32] Marchisio P. Esposito S. Schito G. C. Marchese A. Cavagna R. Principi N. (2002). Nasopharyngeal carriage of *Streptococcus pneumoniae* in healthy children: implications for the use of heptavalent pneumococcal conjugate vaccine. Emerg. Infect. Dis. 8, 479–484. 10.3201/eid0805.010235 11996682 PMC2732490

[B33] Martens P. Worm S. W. Lundgren B. Konradsen H. B. Benfield T. (2004). Serotype-specific mortality from invasive *Streptococcus pneumoniae* disease revisited. BMC Infect. Dis. 4:21. 10.1186/1471-2334-4-21 15228629 PMC455681

[B34] Marthos B. V. Ferri A. L. S. de Figueiredo D. B. Zangirolami T. C. Gonçalves V. M. (2015). Capsular polysaccharide production by *Streptococcus pneumoniae* serotype 1: from strain selection to fed-batch cultivation. Appl. Microbiol. Biotechnol. 99, 10447–10456. 10.1007/s00253-015-6928-z 26298702

[B35] Merieux S. I. Arminjon F. Donikian R. (1981). Procede de purification de polyosydes de streptococcus pneumoniae et vaccin a base de polyosides ainsi purifies. Patent WO1982001995A1. Available online at: https://patents.google.com/patent/WO1982001995A1/fr

[B36] Mitchell T. J. Andrew P. W. Saunders F. K. Smith A. N. Boulnois G. J. (1991). Complement activation and antibody binding by pneumolysin via a region of the toxin homologous to a human acute-phase protein. Mol. Microbiol. 5, 1883–1888. 10.1111/j.1365-2958.1991.tb00812.x 1766369

[B37] Morais V. Suárez N. (2016). Economic evaluation of *Streptococcus pneumoniae* culture media. Am. J. Biochem. Biotechnol. 12, 133–138. 10.3844/ajbbsp.2016.133.138

[B38] Morona J. K. Morona R. Paton J. C. (1997). Characterization of the locus encoding the *Streptococcus pneumoniae* type 19F capsular polysaccharide biosynthetic pathway. Mol. Microbiol. 23, 751–763. 10.1046/j.1365-2958.1997.2551624.x 9157246

[B39] Neeleman C. Geelen S. P. M. Aerts P. C. Daha M. R. Mollnes T. E. Roord J. J.. (1999). Resistance to both complement activation and phagocytosis in type 3 pneumococci is mediated by the binding of complement regulatory protein factor H. Infect. Immun. 67, 4517–4524. 10456894 10.1128/iai.67.9.4517-4524.1999 PMC96772

[B40] Pato T. P. Barbosa A. D. P. R. da Silva Junior J. G. (2006). Purification of capsular polysaccharide from *Neisseria meningitidis* serogroup C by liquid chromatography. J. Chromatogr. B. Analyt. Technol. Biomed. Life Sci. 832, 262–267. 10.1016/j.jchromb.2006.01.008 16469547

[B41] Sharma S. Hanif S. Kumar N. Joshi N. Rana R. Dalal J.. (2015). Rapid processes for purification of capsular polysaccharides from *Neisseria meningitidis* serogroups A and C. Biologicals 43, 383–389. 10.1016/j.biologicals.2015.06.003 26123432

[B42] Suárez N. Fraguas L. F. Texeira E. Massaldi H. Batista-Viera F. Ferreira F. (2001). Production of capsular polysaccharide of *Streptococcus pneumoniae* type 14 and its purification by affinity chromatography. Appl. Environ. Microbiol. 67, 969–971. 10.1128/AEM.67.2.969-971.2001 11157270 PMC92674

[B43] Tanizaki M. M Garcia L. R. Ramos J. B. Leite L. C. C. Hiss H. Furuta J. A. (1996). Purification of meningococcal group C polysaccharide by a procedure suitable for scale-up. J. Microbiol. Methods 27, 19–23. 10.1016/0167-7012(96)00921-9

[B44] Watson D. A. Musher D. M. (1990). Interruption of capsule production in *Streptococcus pneumoniae* serotype 3 by insertion of transposon Tn916. Infect. Immun. 58, 3135–3138. 2167295 10.1128/iai.58.9.3135-3138.1990 PMC313622

[B45] Wattal C. Oberoi J. K. Pruthi P. K. Gupta S. (2007). Nasopharyngeal carriage of *Streptococcus pneumoniae*. Indian J. Pediatr. 74, 905–907. 10.1007/s12098-007-0166-z 17978447

[B46] WHO (2009). Recommendations to Assure the Quality, Safety and Efficacy of Pneumococcal Conjugate Vaccines. Geneva Available online at: http://www.who.int/biologicals/areas/vaccines/pneumo/Pneumo_final_23APRIL_2010.pdf?ua=1

[B47] Wu K. Xu H. Zheng Y. Wang L. Zhang X. Yin Y.. (2016). CpsR, a GntR family regulator, transcriptionally regulates capsular polysaccharide biosynthesis and governs bacterial virulence in *Streptococcus pneumoniae*. Sci. Rep. 6:29255. 10.1038/srep29255 27386955 PMC4937376

[B48] Yother J. (2011). Capsules of *Streptococcus pneumoniae* and other bacteria: paradigms for polysaccharide biosynthesis and regulation. Annu. Rev. Microbiol. 65, 563–581. 10.1146/annurev.micro.62.081307.162944 21721938

[B49] Zafar M. A. Hamaguchi S. Zangari T. Cammer M. Weiser J. N. (2017). Capsule type and amount affect shedding and transmission of *Streptococcus pneumoniae*. MBio 8:e00989–e00917. 10.1128/mBio.00989-17 28830943 PMC5565965

[B50] Zanardo R. T. Ferri A. L. S. Figueiredo D. B. Kraschowetz S. Cabrera-Crespo J. Gonçalves V. M. (2016). Development of a new process for purification of capsular polysaccharide from *Streptococcus pneumoniae* Serotype 14. Braz. J. Chem. Eng. 33, 435–443. 10.1590/0104-6632.20160333s20150140

[B51] Zhang J. Robinson D. (2005). Development of animal-free, protein-free and chemically-defined media for NS0 cell culture. Cytotechnology 48, 59–74. 10.1007/s10616-005-3563-z 19003032 PMC3449720

